# Status report of the blood transfusion services in Myanmar

**DOI:** 10.4103/0973-6247.45258

**Published:** 2009-01

**Authors:** Thida Aung

**Affiliations:** *Director, National, Blood Center, Yangon, Myanmer*

## Introduction

The Myanmar Red Cross Society (MRCS) initiated blood donation activities in 1961, and since then, has been organizing regular blood donation activities. The MRCS supports the ‘national blood and blood product law’ (enacted in January 2003) to save patients lives through blood transfusion of quality assured blood and blood products and to prevent transfusion transmissible infections through the promotion of Voluntary Nonremunerated Blood Donation (VNRBD). Every day, millions of people in the country require blood transfusion. Most transfusions save lives, but there is always a risk of infection from contaminated blood. Safe blood may generally be described as having no trace of viruses, parasites, or other factors that may cause harm to the recipient. A system of regular blood donation from voluntary nonremunerated blood donors is widely recognized as a critical factor in quality blood service delivery. Blood collected from family or replacement donors, especially paid donors, is known to have a higher incidence and prevalence of transfusion-transmissible infections. Blood collected from a voluntary system is safe, and less likely to contain HIV, Hepatitis B or C, or some other infections. A crucial element in ensuring safety is to know as much as possible about the source of donated blood. Voluntary nonremunerated blood donors who chip-in blood regularly are the low-risk donors because they are motivated solely by altruism and have no reason to conceal. This requires the establishment of an effective blood donor program for education, motivation, and recruitment of voluntary blood donors, assessing the suitability of donors, safe blood collection procedures, and high-quality donor care to promote donor retention.

Union of Myanmar, with an area of 676.578 square kilometers, has a total population of 52.17 millions. The population density of the capital is 390/sq km, whereas that of China state is 10/sq km. There are 17 states/divisions, 65 districts, and 325 branches. There are 135 national groups of more than 100 languages. Seventy percent of population resides in rural areas and 30% in urban expanse.

## Blood Transfusion Service in Myanmar

In the year 1899, blood transfusion was initiated at Yangon General Hospital. During 1939 blood transfusion services were formalized and paid blood donation program started. In the year 1945, blood bank facility was established at Yangon General Hospital, which is now known as National Blood Center (NBC). Thereafter, in the year 1962, national blood bank committee was created. Immediately after that, in the year 1963, voluntary blood donation program was formalized.

The NBC is managed directly by the department of health. There are two national blood banks – one is at Yangon General hospital and the other at Mandalay General Hospital – with an annual demand of 180,000 units of blood. There is a nationwide network of 359 hospital-based blood banks with a demand of 200,000 units of blood. There are many voluntary organizations that assist in donor recruitment and blood donation. There are six teaching hospitals, 28 general hospitals, 45 district level hospitals, 19 specialty hospitals, and more than 324 townships and station level hospitals that exercise regular blood transfusions.

## Myanmar Red Cross Society

MRCS, established in 1920, has nationwide network of volunteers across headquarter and its 325 branches. MRCS is having almost 250,000 volunteers working for humanitarian cause throughout the country. Blood donation support was initiated in 1961 with organized Red Cross Volunteers (RCVs) as blood donors. MRCS working committee was created in 2004 and VNRBD program was solemnized to achieve the target.

The objective of MRCS has been to improve the amount of safe blood available through increased recruitment and retention of voluntary nonremunerated blood donors in coordination and collaboration with NBC. MRCS commitment to VNRBD is exhibited through the formation of a working committee, training over 200 RCVs in blood donor recruitment, promotion of youth blood donor groups, supply of supportive materials such as cold storage facilities to the national blood service, and general community awareness campaigns. MRCS is a regular member of South East Asia Regional Workshops and hosted the fourth regional workshop in 2006.

## Components of Myanmar Red Cross Society Voluntary Nonremunerated Blood Donation Program

### Advocacy and public awareness

Is mainly via promotion of healthy lifestyle and identification of the low-risk groups as potential blood donors. Public awareness is created by meeting with local administrative authorities and communities, usually at the states/divisional level and branches, and disseminating on the value of volunteer blood donors. Efforts are made to explain the importance of safe blood supply and to promote healthy lifestyle among general public. Also, they are explained about the risk factors and the transmission of transfusion-transmissible infections. These type of meetings help identify the low-risk groups as potential blood donors.

### Donor motivation, recruitment, and retention

Various methodologies are applied to promote voluntary blood donor motivation recruitment. These activities are carried out in coordination of MRCS and NBC. Various awareness programs in the community are carried out to explain the importance of voluntary blood donations by using different means.

As moving toward 100% voluntary blood donation, all the potential donors will be screened for TTI, the voluntary donors will be retained to phaseout the replacement donors, and community awareness campaigns will reach the community.

Methodology for voluntary blood donor recruitment

**Figure 1:**
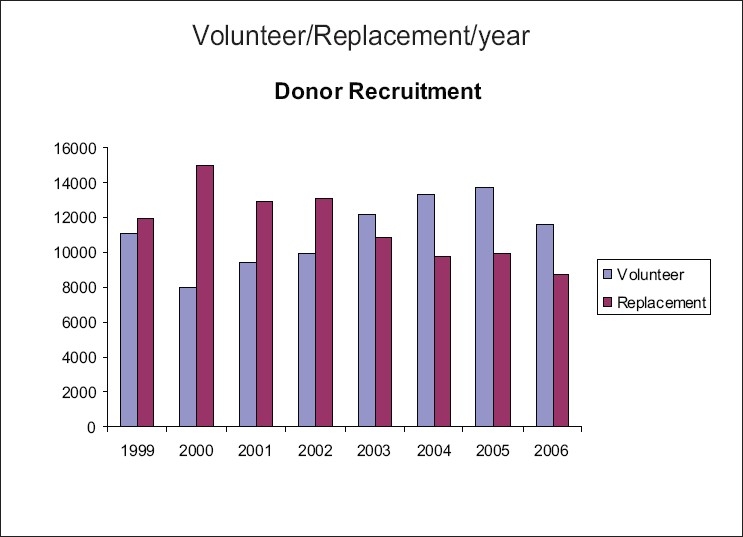
Donor recruitment from 1999 to 2006
- MRCS contributes to improve the amount of safe blood available in Myanmar through increased recruitment and retention of voluntary nonremunerated blood donors. It is clearly reflected in the year-wise TTI positive data as shown in Figure 2.

- Effect of continuous efforts of MRCS and MBC toward promotion of VNRBD is detailed in different graphs. The graph in figure 1 shows volunteer/replacement pattern of blood donation changing year-by-year because of community education.

**Figure:**
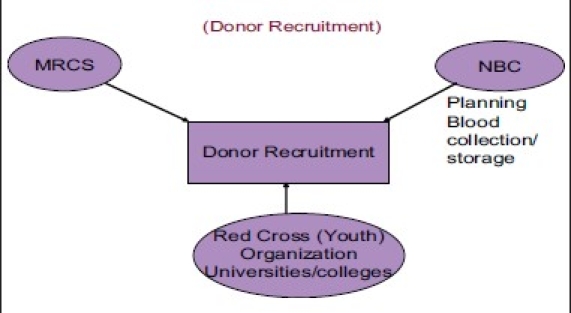
Donor recruitment structure in Myanmar

**Figure 2:**
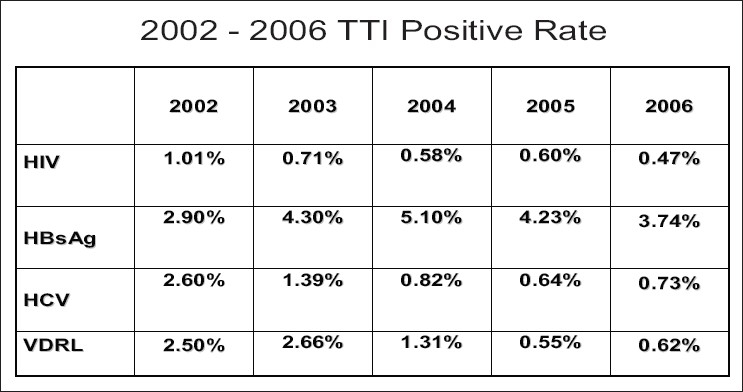
TTI positivity in blood donors

**Figure 3:**
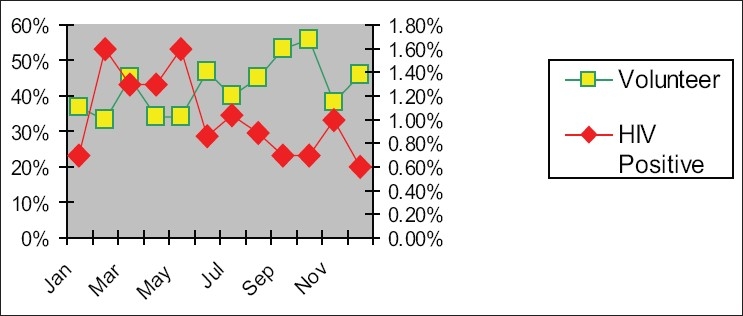
Association of HIV positivity in blood donors (2002)

**Figure 4:**
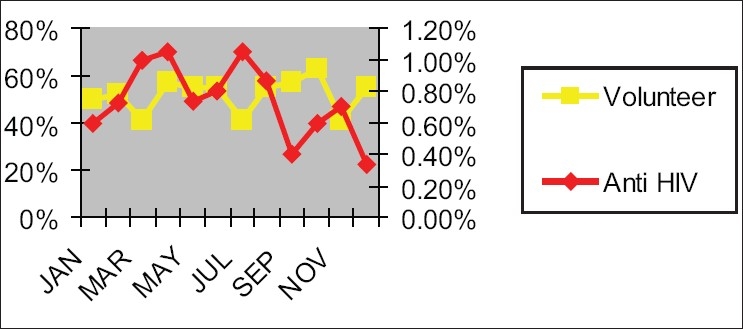
Association of HIV positivity in blood donors (2003)

**Figure 5:**
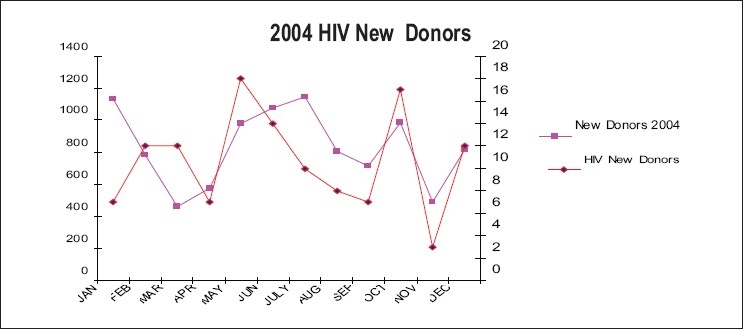
Association of HIV positivity in new blood donors (2004)

**Figure 6:**
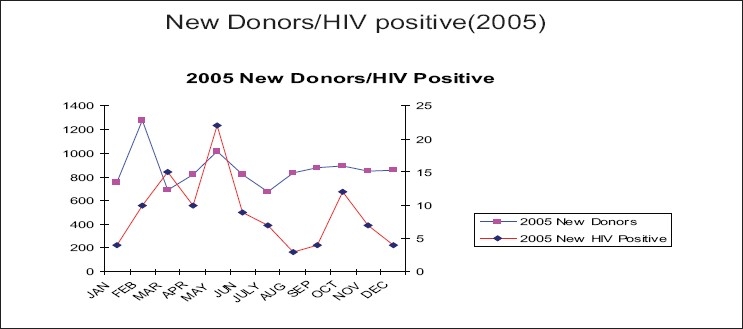
Association of HIV positivity in new blood donors (2005)

Graphs in Figures 3–6 show the incidence of volunteer/incidence of HIV between 2002 and 2005. It also points out that HIV rate decreased with the increased regular blood donors and HIV rate increased with the reduced amount of regular blood donors.

### Training of trainers

Four days training is given to RCVs, using “making a difference” module, since 2004. A total of 216 RCVs have been trained thus far. In the year 2006, students from universities and colleges were trained for youth-based recruitment team. School children were also made aware of life saving properties of blood via a chapter on the same in their Red Cross syllabus. Community based first aid program also had discussion on the facts of safe blood. The training done for RCVs on blood donor recruitment from 2004-2006 is presented in Table 1.

**Table 1:** Training of trainers

1 TOT on RCV blood donor recruitment 30 participants	December 2004 (15 tsps. From Yangon Division)
2 TOT on RCV blood donor recruitment 30 participant	April 2005 (15 tsps. From Yangon Division)
3 National workshop on voluntary blood donor recruitment program 36 participants	February 2005 (17 States/Divisions)
4 TOT on RCV blood donor recruitment 30 participants	June 2005 (7 tsps. From Mandalay Division)
5 TOT on RCV blood donor recruitment 30 participants	February 2006 (15 tsps. From Yangon Division)
6 TOT on youth blood donor recruitment 30 participants	June 2006 (13 Universities/Colleges from Yangon Division and Moe-nyo tsp: from Bago (West)
7 Workshop on developing voluntary blood donor program for blood safety	December 2006 (10 Uni: RC + 9 branches + NCB)

### Celebration of World Blood Donor Day

**Figure 7:**
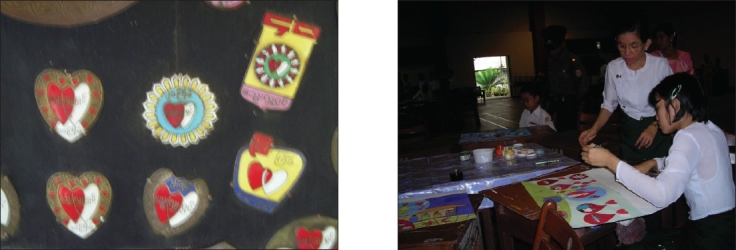
World Blood Donor day poster competition

**Figure 8:**
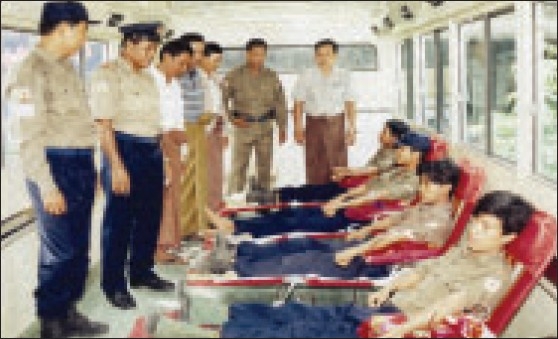
Mass Blood Donation Campaign

**Figure 9:**
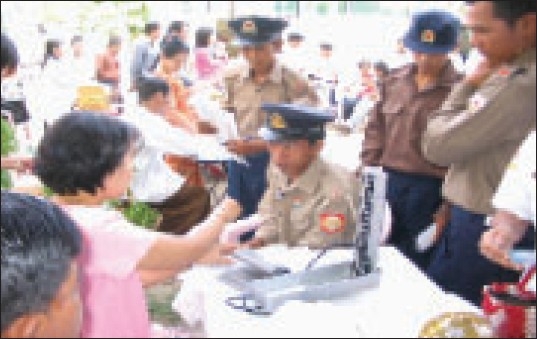
Screening before blood donation

**Figure 10:**
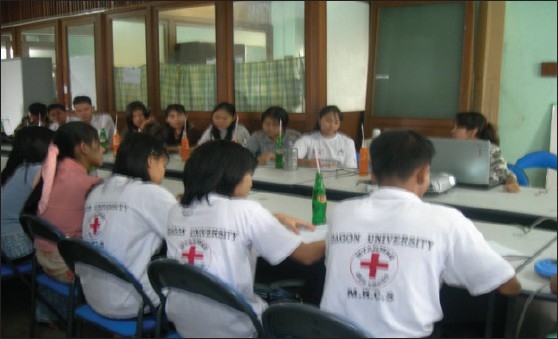
RCV youth team

Since 2004, WBDD is being celebrated every year in the month of June by motivating people for voluntary blood donation. Mass blood donation on this day is done with a reminder letter for the blood recruiters and RCVs. Awards and recognition badges [Figure 7–10] and appreciation certificates are presented. The National Blood Donor day in the month of December is also celebrated in a similar manner.

### World blood donor day poster competition

There was a poster competition in 2007 on WBDD, where 77 students participated and the painting took nearly three hours to complete. The winners were given prizes on WBDD. The students went to the NBC where they could watch the donors being screened with a questionnaire, precounseled, and their body weight and hemoglobin being checked.

The outstanding blood donors on the WBDD were awarded on the Independence day (January 4). There was a television program on the importance of blood donation to motivate potential as well as regular donors to donate blood for the needy.

Transportation charges were given to mass blood donation group and NBC supplied with refreshments and iron supplements.

### Social mobilization by mass education and motivation campaign

MRCS is continuously involved in educating the society by mass education programs and motivational activities. MRCS is arranging voluntary blood donation camps where different classes of society come forward to donate blood.

MRCS youth-based team is also involved in MRCS VNRBD program. Youths aged 16-25 years form 23% of the total volunteer blood donor population. The youth team is designed to inform and educate youth on the importance of blood donation in saving lives as well as the importance of being blood donors.

A group of young people below 25 years, members of universities/colleges RC youth teams, recruit new RC youth members and youth blood donors and also promote healthy lifestyle and safe blood donation among the youth groups.

### Challenges for the Myanmar Red Cross Society Voluntary in Nonremunerated Blood Donation Program

1. Poor systematic organization of donor recruitment in the branches, although they are donating blood in emergencies as living blood donors
2. Community beliefs and fears concerning blood donation still present and there is a need for the donor education program, especially in schools
3. Limited understanding by the community of the ongoing need for blood
4. Minimal reporting of blood donor activities where the standardized format is not ready
5. Community awareness including schools still needs to be scaled up
6. Review and coordination meeting identified that there are some gaps like weakness in communication from the branches/state/division to the N.S/NBC/hospital based blood bank and vice versa

To overcome these challenges MRCS has developed certain future directions:

- Systematic implementation of MRCS VNRBR program
- Program guidelines to be standardized
- Standardized training module guide for training of trainers
- Expand recruiters training of trainers in states and divisions for nationwide implementation
- Develop database for RC donor registry and follow-up
- Develop reporting system
- Improve and institutionalize monitoring and evaluation system
- Contingency planning for emergency blood donation support
- Increase blood donor recruitment and blood donation activities to be planned in collaboration with other partners and department of health
- Sustain coordination and collaboration with NBC and local hospitals
- NBC regular meetings with MRCS blood working groups
- Increase RC youth donor teams in colleges and universities
- Intensify community awareness and IEC distribution
- Formalized awards, incentives, and recognition system for RCVs blood donors associations

## Conclusion

MRCS in coordination with and support by NBC (National Health Authorities) for a comprehensive blood program, having a strong base of voluntary, nonremunerated blood donors trying to phaseout family and replacement donation and implementing quality systems throughout the blood chain, from the education of blood donors to the transfusion of blood to the patient.

The National Society is still preparing a standard training package for blood recruiter, roles and responsibilities, recognition badges, and data registry for all branches.
